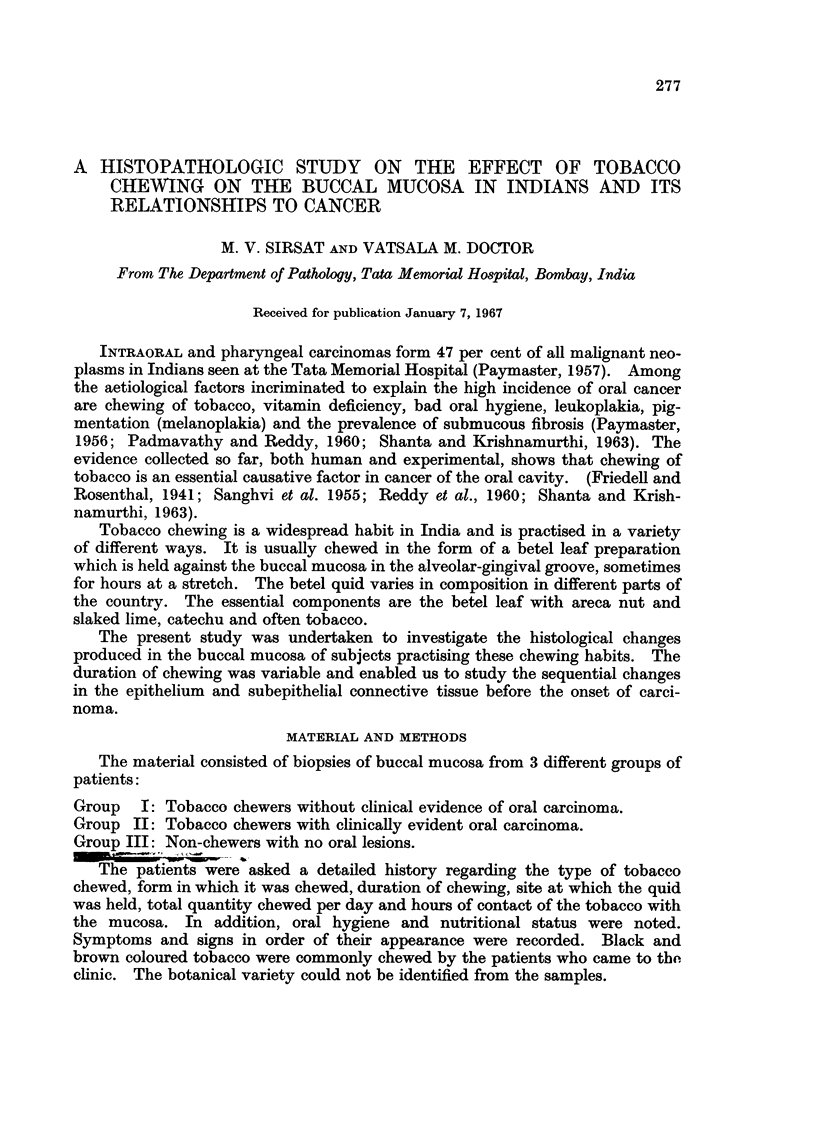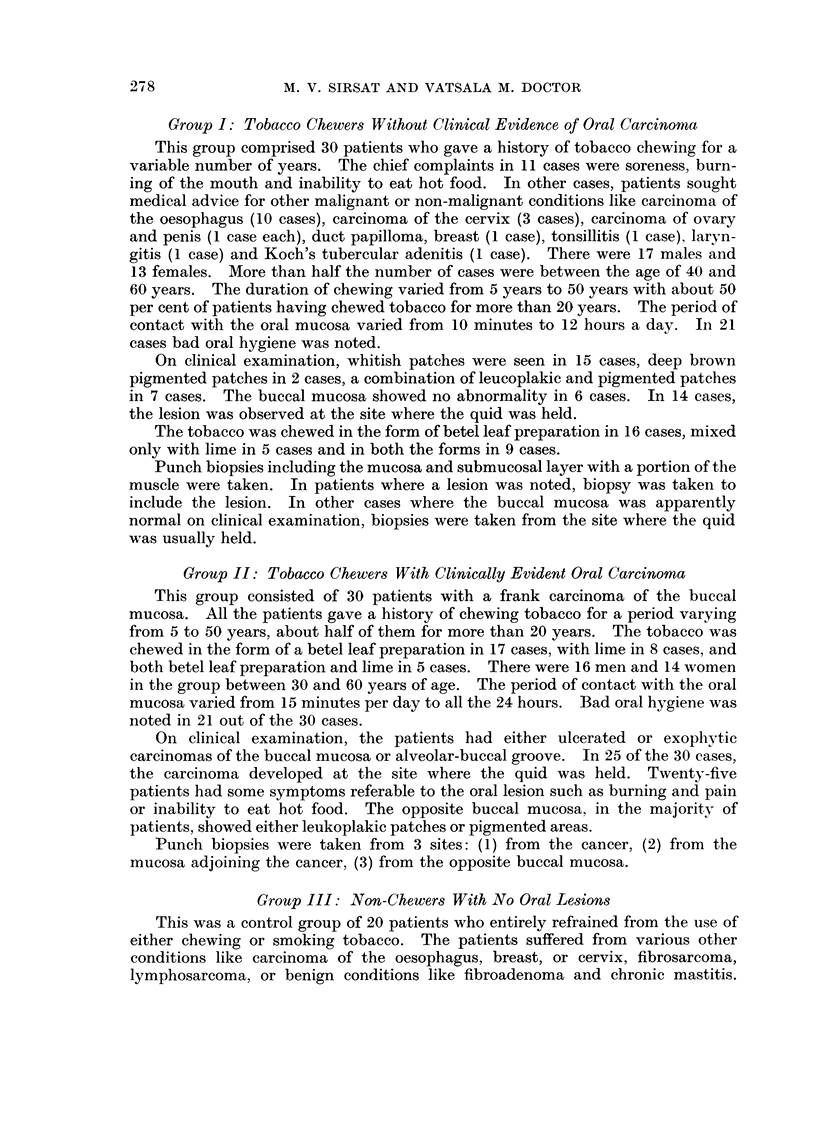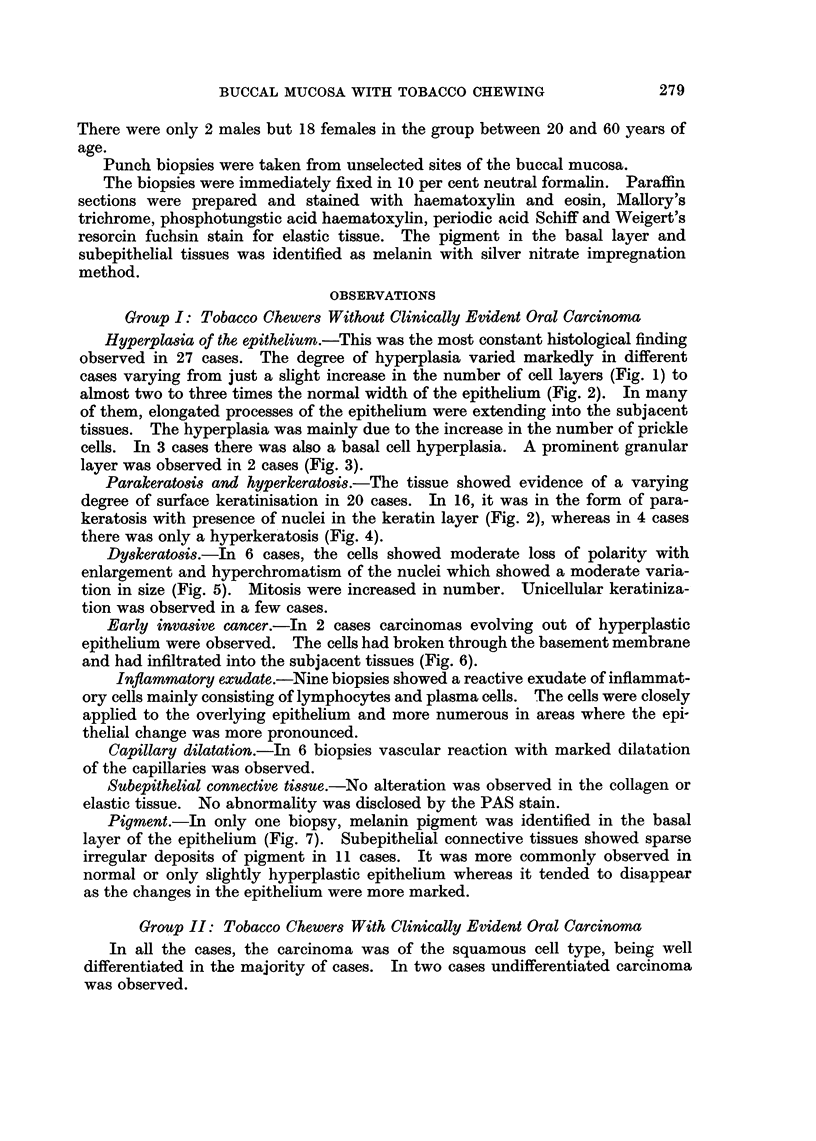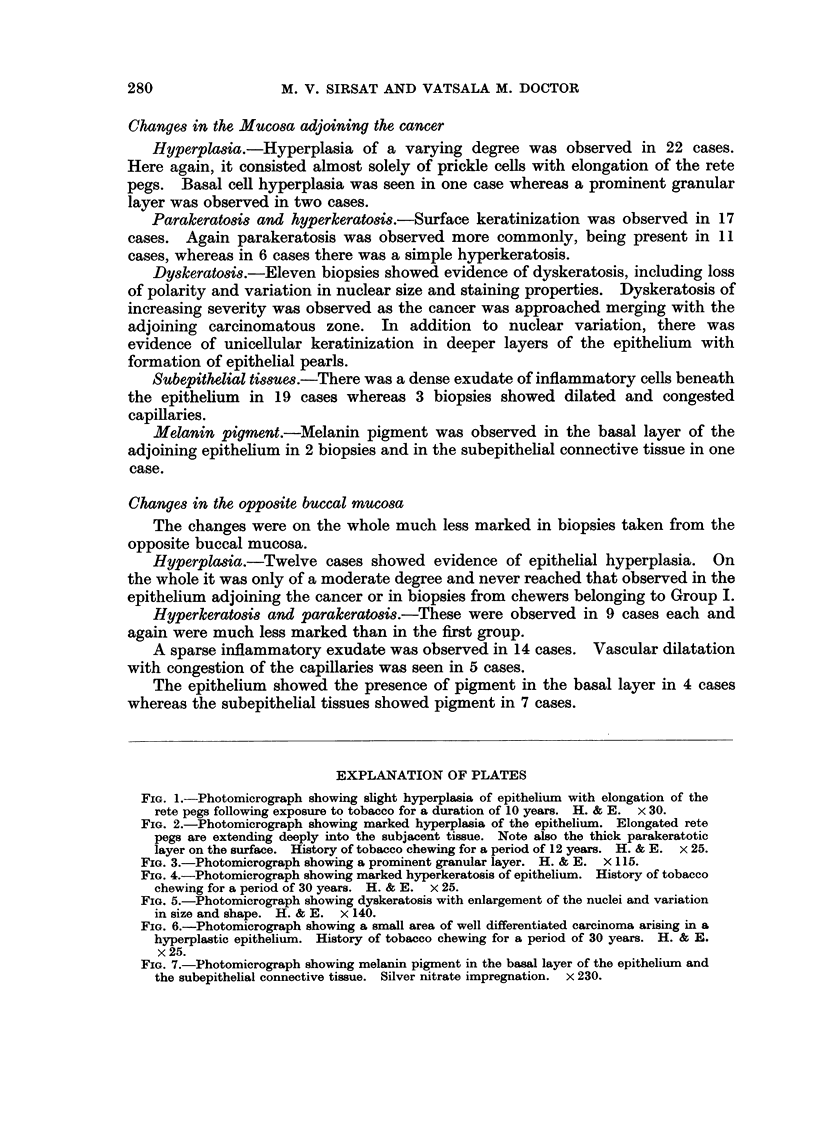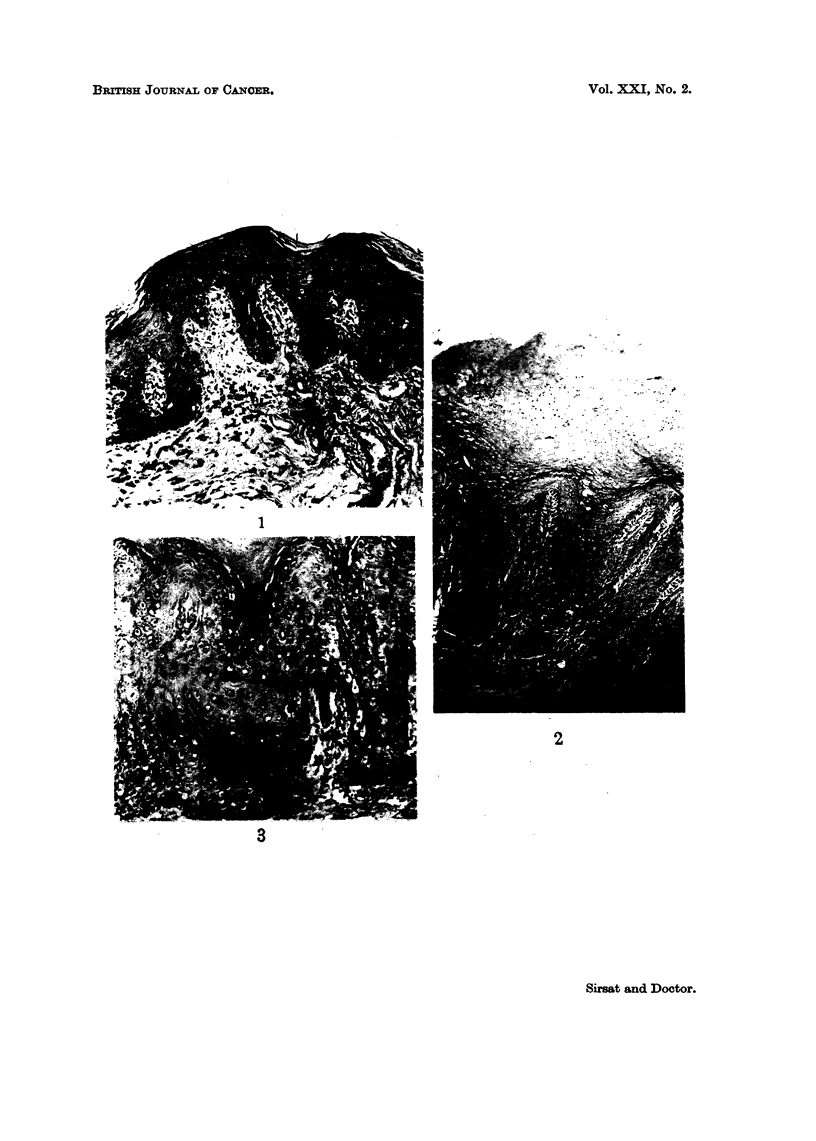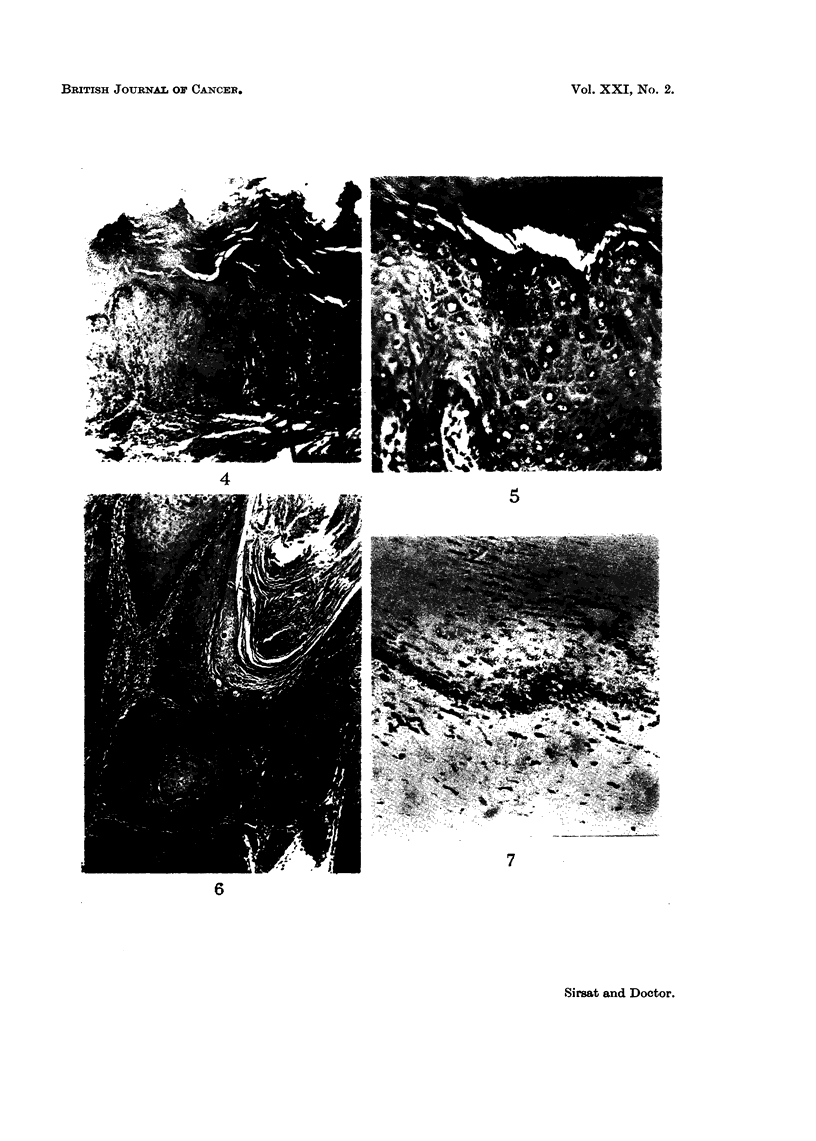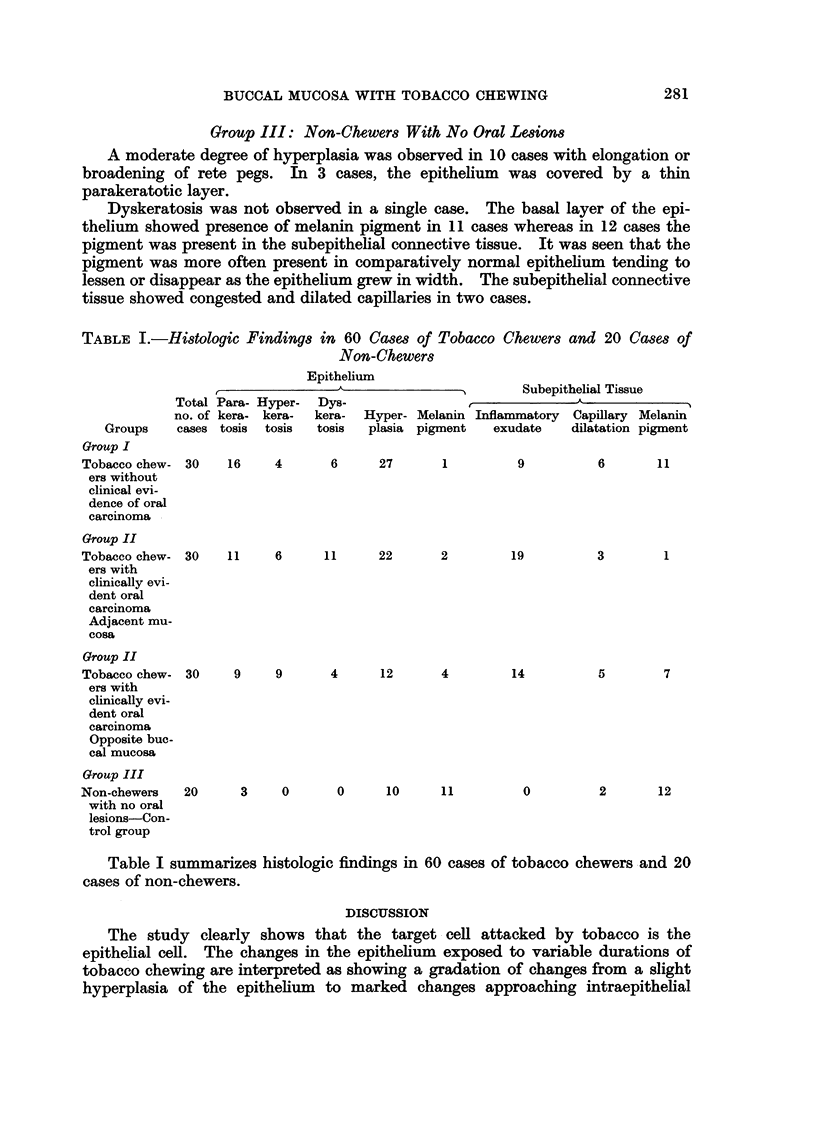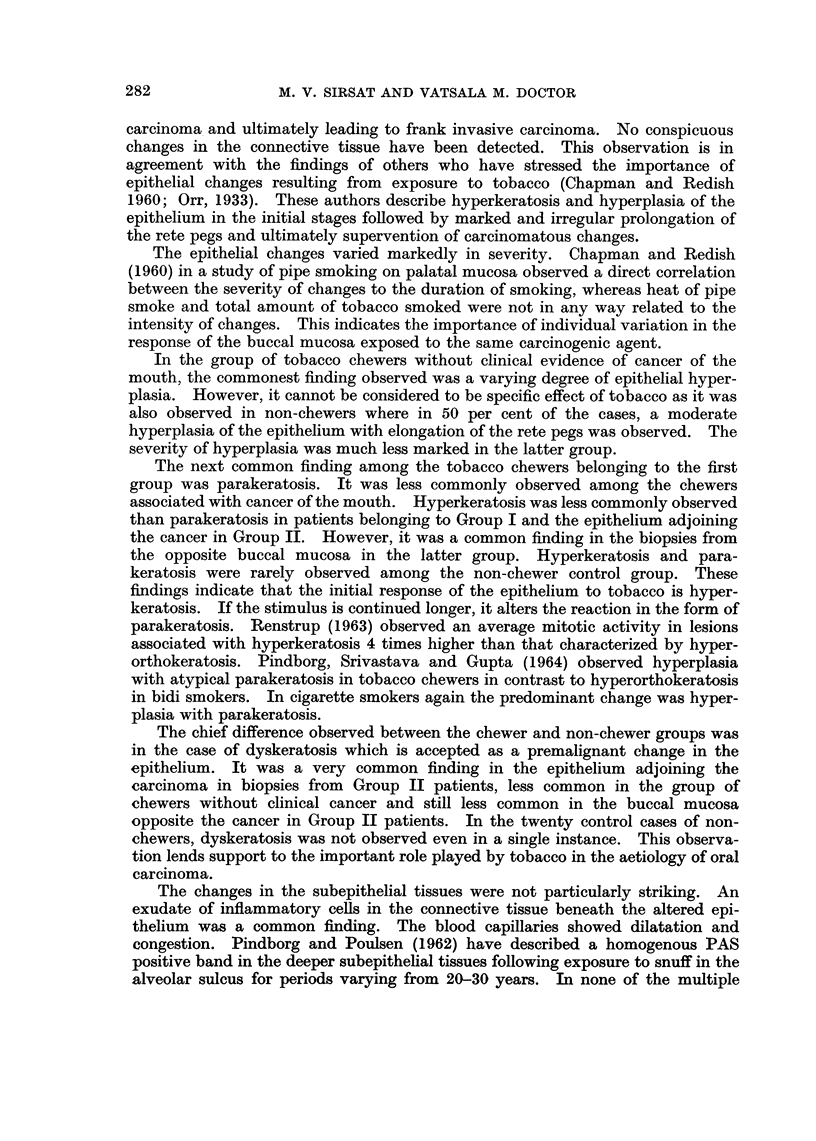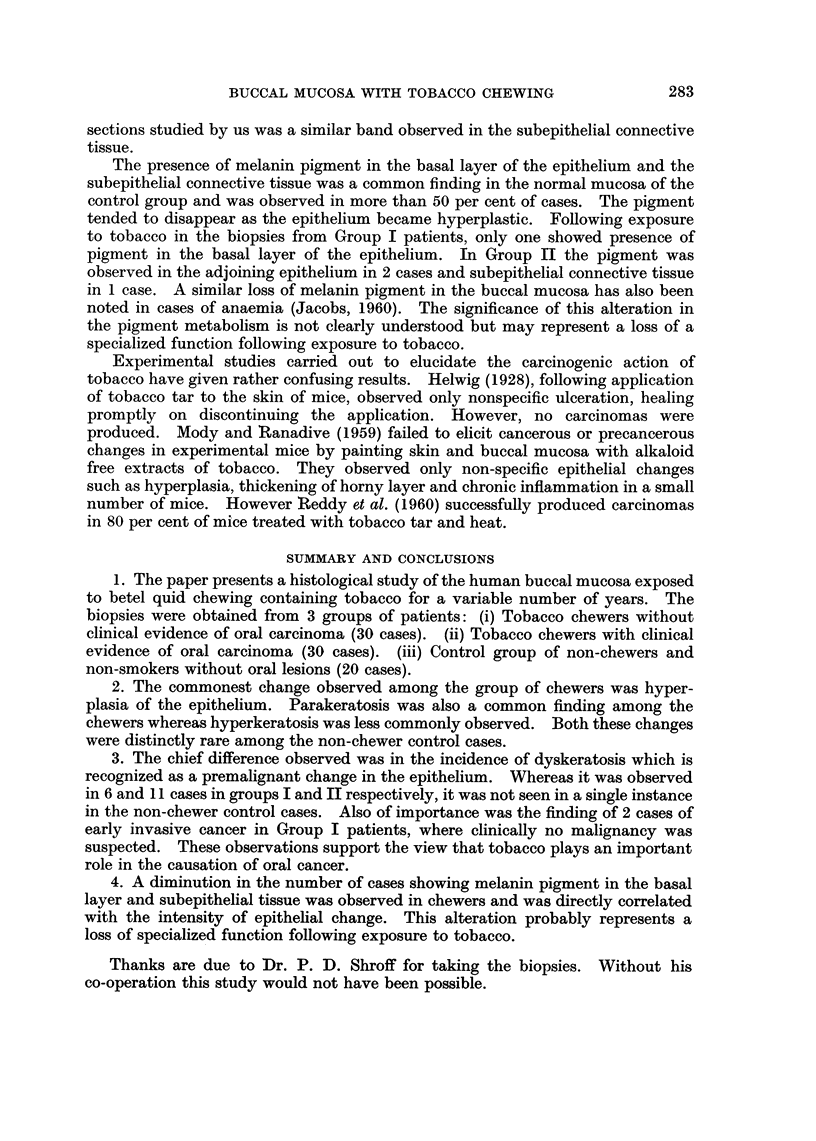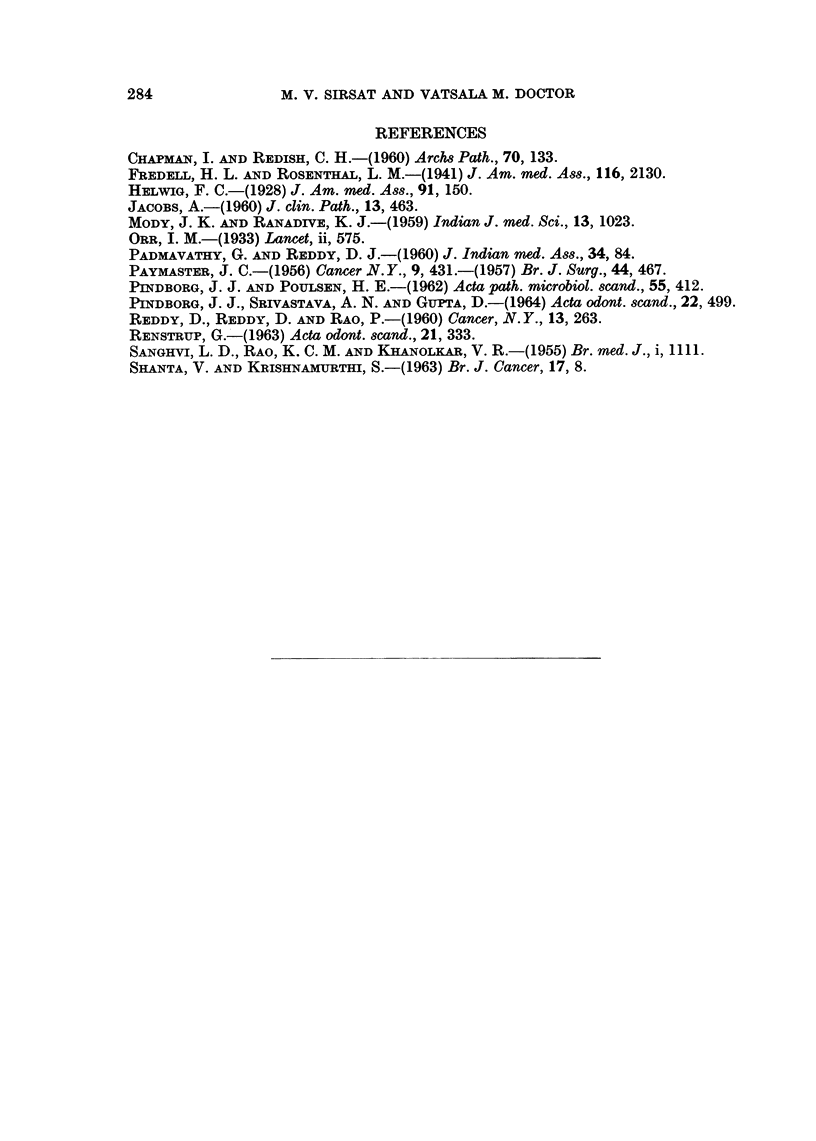# A histopathologic study on the effect of tobacco chewing on the buccal mucosa in Indians and its relationships to cancer.

**DOI:** 10.1038/bjc.1967.30

**Published:** 1967-06

**Authors:** M. V. Sirsat, V. M. Doctor

## Abstract

**Images:**


### A HISTOPATHOLOGIC STUDY ON THE EFFECT OF TOBACCO CHEWING ON THE BUCCAL MUCOSA IN INDIANS AND ITS RELATIONSHIPS TO CANCER

M. V. SIRSAT AND VATSALA M. DOCTOR

*From The Department of Pathology, Tata Memorial Hospital, Bombay, India*

Received for publication January 7, 1967

INTRAORAL and pharyngeal carcinomas form 47 per cent of all malignant neoplasms in Indians seen at the Tata Memorial Hospital (Paymaster, 1957). Among the aetiological factors incriminated to explain the high incidence of oral cancer are chewing of tobacco, vitamin deficiency, bad oral hygiene, leukoplakia, pigmentation (melanoplakia) and the prevalence of submucous fibrosis (Paymaster, 1956; Padmavathy and Reddy, 1960; Shanta and Krishnamurthi, 1963). The evidence collected so far, both human and experimental, shows that chewing of tobacco is an essential causative factor in cancer of the oral cavity. (Friedell and Rosenthal, 1941; Sanghvi *et al.* 1955; Reddy *et al.*, 1960; Shanta and Krishnamurthi, 1963).

Tobacco chewing is a widespread habit in India and is practised in a variety of different ways. It is usually chewed in the form of a betel leaf preparation which is held against the buccal mucosa in the alveolar-gingival groove, sometimes for hours at a stretch. The betel quid varies in composition in different parts of the country. The essential components are the betel leaf with areca nut and slaked lime, catechu and often tobacco.

The present study was undertaken to investigate the histological changes produced in the buccal mucosa of subjects practising these chewing habits. The duration of chewing was variable and enabled us to study the sequential changes in the epithelium and subepithelial connective tissue before the onset of carcinoma.

### MATERIAL AND METHODS

The material consisted of biopsies of buccal mucosa from 3 different groups of patients:

Group I: Tobacco chewers without clinical evidence of oral carcinoma.
Group II: Tobacco chewers with clinically evident oral carcinoma.
Group III: Non-chewers with no oral lesions.

The patients were asked a detailed history regarding the type of tobacco chewed, form in which it was chewed, duration of chewing, site at which the quid was held, total quantity chewed per day and hours of contact of the tobacco with the mucosa. In addition, oral hygiene and nutritional status were noted. Symptoms and signs in order of their appearance were recorded. Black and brown coloured tobacco were commonly chewed by the patients who came to the clinic. The botanical variety could not be identified from the samples.

*Group I: Tobacco Chewers Without Clinical Evidence of Oral Carcinoma*

This group comprised 30 patients who gave a history of tobacco chewing for a variable number of years. The chief complaints in 11 cases were soreness, burning of the mouth and inability to eat hot food. In other cases, patients sought medical advice for other malignant or non-malignant conditions like carcinoma of the oesophagus (10 cases), carcinoma of the cervix (3 cases), carcinoma of ovary and penis (1 case each), duct papilloma, breast (1 case), tonsillitis (1 case), laryngitis (1 case) and Koch's tubercular adenitis (1 case). There were 17 males and 13 females. More than half the number of cases were between the age of 40 and 60 years. The duration of chewing varied from 5 years to 50 years with about 50 per cent of patients having chewed tobacco for more than 20 years. The period of contact with the oral mucosa varied from 10 minutes to 12 hours a day. In 21 cases bad oral hygiene was noted.

On clinical examination, whitish patches were seen in 15 cases, deep brown pigmented patches in 2 cases, a combination of leucoplakic and pigmented patches in 7 cases. The buccal mucosa showed no abnormality in 6 cases. In 14 cases, the lesion was observed at the site where the quid was held.

The tobacco was chewed in the form of betel leaf preparation in 16 cases, mixed only with lime in 5 cases and in both the forms in 9 cases.

Punch biopsies including the mucosa and submucosal layer with a portion of the muscle were taken. In patients where a lesion was noted, biopsy was taken to include the lesion. In other cases where the buccal mucosa was apparently normal on clinical examination, biopsies were taken from the site where the quid was usually held.

*Group II: Tobacco Chewers With Clinically Evident Oral Carcinoma*

This group consisted of 30 patients with a frank carcinoma of the buccal mucosa. All the patients gave a history of chewing tobacco for a period varying from 5 to 50 years, about half of them for more than 20 years. The tobacco was chewed in the form of a betel leaf preparation in 17 cases, with lime in 8 cases, and both betel leaf preparation and lime in 5 cases. There were 16 men and 14 women in the group between 30 and 60 years of age. The period of contact with the oral mucosa varied from 15 minutes per day to all the 24 hours. Bad oral hygiene was noted in 21 out of the 30 cases.

On clinical examination, the patients had either ulcerated or exophytic carcinomas of the buccal mucosa or alveolar-buccal groove. In 25 of the 30 cases, the carcinoma developed at the site where the quid was held. Twenty-five patients had some symptoms referable to the oral lesion such as burning and pain or inability to eat hot food. The opposite buccal mucosa, in the majority of patients, showed either leukoplakic patches or pigmented areas.

Punch biopsies were taken from 3 sites: (1) from the cancer, (2) from the mucosa adjoining the cancer, (3) from the opposite buccal mucosa.

*Group III: Non-Chewers With No Oral Lesions*

This was a control group of 20 patients who entirely refrained from the use of either chewing or smoking tobacco. The patients suffered from various other conditions like carcinoma of the oesophagus, breast, or cervix, fibrosarcoma, lymphosarcoma, or benign conditions like fibroadenoma and chronic mastitis.

There were only 2 males but 18 females in the group between 20 and 60 years of age.

Punch biopsies were taken from unselected sites of the buccal mucosa.

The biopsies were immediately fixed in 10 per cent neutral formalin. Paraffin sections were prepared and stained with haematoxylin and eosin, Mallory's trichrome, phosphotungstic acid haematoxylin, periodic acid Schiff and Weigert's resorcin fuchsin stain for elastic tissue. The pigment in the basal layer and subepithelial tissues was identified as melanin with silver nitrate impregnation method.

### OBSERVATIONS

### *Group I: Tobacco Chewers Without Clinically Evident Oral Carcinoma*

*Hyperplasia of the epithelium.*—This was the most constant histological finding observed in 27 cases. The degree of hyperplasia varied markedly in different cases varying from just a slight increase in the number of cell layers (Fig. 1) to almost two to three times the normal width of the epithelium (Fig. 2). In many of them, elongated processes of the epithelium were extending into the subjacent tissues. The hyperplasia was mainly due to the increase in the number of prickle cells. In 3 cases there was also a basal cell hyperplasia. A prominent granular layer was observed in 2 cases (Fig. 3).

*Parakeratosis and hyperkeratosis.*—The tissue showed evidence of a varying degree of surface keratinisation in 20 cases. In 16, it was in the form of parakeratosis with presence of nuclei in the keratin layer (Fig. 2), whereas in 4 cases there was only a hyperkeratosis (Fig. 4).

*Dyskeratosis.*—In 6 cases, the cells showed moderate loss of polarity with enlargement and hyperchromatism of the nuclei which showed a moderate variation in size (Fig. 5). Mitosis were increased in number. Unicellular keratinization was observed in a few cases.

*Early invasive cancer.*—In 2 cases carcinomas evolving out of hyperplastic epithelium were observed. The cells had broken through the basement membrane and had infiltrated into the subjacent tissues (Fig. 6).

*Inflammatory exudate.*—Nine biopsies showed a reactive exudate of inflammatory cells mainly consisting of lymphocytes and plasma cells. The cells were closely applied to the overlying epithelium and more numerous in areas where the epithelial change was more pronounced.

*Capillary dilatation.*—In 6 biopsies vascular reaction with marked dilatation of the capillaries was observed.

*Subepithelial connective tissue.*—No alteration was observed in the collagen or elastic tissue. No abnormality was disclosed by the PAS stain.

*Pigment.*—In only one biopsy, melanin pigment was identified in the basal layer of the epithelium (Fig. 7). Subepithelial connective tissues showed sparse irregular deposits of pigment in 11 cases. It was more commonly observed in normal or only slightly hyperplastic epithelium whereas it tended to disappear as the changes in the epithelium were more marked.

### *Group II: Tobacco Chewers With Clinically Evident Oral Carcinoma*

In all the cases, the carcinoma was of the squamous cell type, being well differentiated in the majority of cases. In two cases undifferentiated carcinoma was observed.

*Changes in the Mucosa adjoining the cancer*

*Hyperplasia.*—Hyperplasia of a varying degree was observed in 22 cases. Here again, it consisted almost solely of prickle cells with elongation of the rete pegs. Basal cell hyperplasia was seen in one case whereas a prominent granular layer was observed in two cases.

*Parakeratosis and hyperkeratosis.*—Surface keratinization was observed in 17 cases. Again parakeratosis was observed more commonly, being present in 11 cases, whereas in 6 cases there was a simple hyperkeratosis.

*Dyskeratosis.*—Eleven biopsies showed evidence of dyskeratosis, including loss of polarity and variation in nuclear size and staining properties. Dyskeratosis of increasing severity was observed as the cancer was approached merging with the adjoining carcinomatous zone. In addition to nuclear variation, there was evidence of unicellular keratinization in deeper layers of the epithelium with formation of epithelial pearls.

*Subepithelial tissues.*—There was a dense exudate of inflammatory cells beneath the epithelium in 19 cases whereas 3 biopsies showed dilated and congested capillaries.

*Melanin pigment.*—Melanin pigment was observed in the basal layer of the adjoining epithelium in 2 biopsies and in the subepithelial connective tissue in one case.

*Changes in the opposite buccal mucosa*

The changes were on the whole much less marked in biopsies taken from the opposite buccal mucosa.

*Hyperplasia.*—Twelve cases showed evidence of epithelial hyperplasia. On the whole it was only of a moderate degree and never reached that observed in the epithelium adjoining the cancer or in biopsies from chewers belonging to Group I.

*Hyperkeratosis and parakeratosis.*—These were observed in 9 cases each and again were much less marked than in the first group.

A sparse inflammatory exudate was observed in 14 cases. Vascular dilatation with congestion of the capillaries was seen in 5 cases.

The epithelium showed the presence of pigment in the basal layer in 4 cases whereas the subepithelial tissues showed pigment in 7 cases.

---

### EXPLANATION OF PLATES

FIG. 1.—Photomicrograph showing slight hyperplasia of epithelium with elongation of the rete pegs following exposure to tobacco for a duration of 10 years. H. & E. ×30.

FIG. 2.—Photomicrograph showing marked hyperplasia of the epithelium. Elongated rete pegs are extending deeply into the subjacent tissue. Note also the thick parakeratotic layer on the surface. History of tobacco chewing for a period of 12 years. H. & E. ×25.

FIG. 3.—Photomicrograph showing a prominent granular layer. H. & E. ×115.

FIG. 4.—Photomicrograph showing marked hyperkeratosis of epithelium. History of tobacco chewing for a period of 30 years. H. & E. ×25.

FIG. 5.—Photomicrograph showing dyskeratosis with enlargement of the nuclei and variation in size and shape. H. & E. ×140.

FIG. 6.—Photomicrograph showing a small area of well differentiated carcinoma arising in a hyperplastic epithelium. History of tobacco chewing for a period of 30 years. H. & E. ×25.

FIG. 7.—Photomicrograph showing melanin pigment in the basal layer of the epithelium and the subepithelial connective tissue. Silver nitrate impregnation. ×230.

1

2

3

Sirsat and Doctor.

4

5

6

7

Sirsat and Doctor.

*Group III: Non-Chewers With No Oral Lesions*

A moderate degree of hyperplasia was observed in 10 cases with elongation or broadening of rete pegs. In 3 cases, the epithelium was covered by a thin parakeratotic layer.

Dyskeratosis was not observed in a single case. The basal layer of the epithelium showed presence of melanin pigment in 11 cases whereas in 12 cases the pigment was present in the subepithelial connective tissue. It was seen that the pigment was more often present in comparatively normal epithelium tending to lessen or disappear as the epithelium grew in width. The subepithelial connective tissue showed congested and dilated capillaries in two cases.

TABLE I.—*Histologic Findings in 60 Cases of Tobacco Chewers and 20 Cases of Non-Chewers*

| Groups | Total no. of cases | Epithelium: Parakeratosis | Hyperkeratosis | Dyskeratosis | Hyperplasia | Melanin pigment | Subepithelial Tissue: Inflammatory exudate | Capillary dilatation | Melanin pigment |
|---|---|---|---|---|---|---|---|---|---|
| *Group I* | | | | | | | | | |
| Tobacco chewers without clinical evidence of oral carcinoma | 30 | 16 | 4 | 6 | 27 | 1 | 9 | 6 | 11 |
| *Group II* | | | | | | | | | |
| Tobacco chewers with clinically evident oral carcinoma Adjacent mucosa | 30 | 11 | 6 | 11 | 22 | 2 | 19 | 3 | 1 |
| *Group II* | | | | | | | | | |
| Tobacco chewers with clinically evident oral carcinoma Opposite buccal mucosa | 30 | 9 | 9 | 4 | 12 | 4 | 14 | 5 | 7 |
| *Group III* | | | | | | | | | |
| Non-chewers with no oral lesions—Control group | 20 | 3 | 0 | 0 | 10 | 11 | 0 | 2 | 12 |

Table I summarizes histologic findings in 60 cases of tobacco chewers and 20 cases of non-chewers.

### DISCUSSION

The study clearly shows that the target cell attacked by tobacco is the epithelial cell. The changes in the epithelium exposed to variable durations of tobacco chewing are interpreted as showing a gradation of changes from a slight hyperplasia of the epithelium to marked changes approaching intraepithelial carcinoma and ultimately leading to frank invasive carcinoma. No conspicuous changes in the connective tissue have been detected. This observation is in agreement with the findings of others who have stressed the importance of epithelial changes resulting from exposure to tobacco (Chapman and Redish 1960; Orr, 1933). These authors describe hyperkeratosis and hyperplasia of the epithelium in the initial stages followed by marked and irregular prolongation of the rete pegs and ultimately supervention of carcinomatous changes.

The epithelial changes varied markedly in severity. Chapman and Redish (1960) in a study of pipe smoking on palatal mucosa observed a direct correlation between the severity of changes to the duration of smoking, whereas heat of pipe smoke and total amount of tobacco smoked were not in any way related to the intensity of changes. This indicates the importance of individual variation in the response of the buccal mucosa exposed to the same carcinogenic agent.

In the group of tobacco chewers without clinical evidence of cancer of the mouth, the commonest finding observed was a varying degree of epithelial hyperplasia. However, it cannot be considered to be specific effect of tobacco as it was also observed in non-chewers where in 50 per cent of the cases, a moderate hyperplasia of the epithelium with elongation of the rete pegs was observed. The severity of hyperplasia was much less marked in the latter group.

The next common finding among the tobacco chewers belonging to the first group was parakeratosis. It was less commonly observed among the chewers associated with cancer of the mouth. Hyperkeratosis was less commonly observed than parakeratosis in patients belonging to Group I and the epithelium adjoining the cancer in Group II. However, it was a common finding in the biopsies from the opposite buccal mucosa in the latter group. Hyperkeratosis and parakeratosis were rarely observed among the non-chewer control group. These findings indicate that the initial response of the epithelium to tobacco is hyperkeratosis. If the stimulus is continued longer, it alters the reaction in the form of parakeratosis. Renstrup (1963) observed an average mitotic activity in lesions associated with hyperkeratosis 4 times higher than that characterized by hyperorthokeratosis. Pindborg, Srivastava and Gupta (1964) observed hyperplasia with atypical parakeratosis in tobacco chewers in contrast to hyperorthokeratosis in bidi smokers. In cigarette smokers again the predominant change was hyperplasia with parakeratosis.

The chief difference observed between the chewer and non-chewer groups was in the case of dyskeratosis which is accepted as a premalignant change in the epithelium. It was a very common finding in the epithelium adjoining the carcinoma in biopsies from Group II patients, less common in the group of chewers without clinical cancer and still less common in the buccal mucosa opposite the cancer in Group II patients. In the twenty control cases of non-chewers, dyskeratosis was not observed even in a single instance. This observation lends support to the important role played by tobacco in the aetiology of oral carcinoma.

The changes in the subepithelial tissues were not particularly striking. An exudate of inflammatory cells in the connective tissue beneath the altered epithelium was a common finding. The blood capillaries showed dilatation and congestion. Pindborg and Poulsen (1962) have described a homogenous PAS positive band in the deeper subepithelial tissues following exposure to snuff in the alveolar sulcus for periods varying from 20–30 years. In none of the multiple sections studied by us was a similar band observed in the subepithelial connective tissue.

The presence of melanin pigment in the basal layer of the epithelium and the subepithelial connective tissue was a common finding in the normal mucosa of the control group and was observed in more than 50 per cent of cases. The pigment tended to disappear as the epithelium became hyperplastic. Following exposure to tobacco in the biopsies from Group I patients, only one showed presence of pigment in the basal layer of the epithelium. In Group II the pigment was observed in the adjoining epithelium in 2 cases and subepithelial connective tissue in 1 case. A similar loss of melanin pigment in the buccal mucosa has also been noted in cases of anaemia (Jacobs, 1960). The significance of this alteration in the pigment metabolism is not clearly understood but may represent a loss of a specialized function following exposure to tobacco.

Experimental studies carried out to elucidate the carcinogenic action of tobacco have given rather confusing results. Helwig (1928), following application of tobacco tar to the skin of mice, observed only nonspecific ulceration, healing promptly on discontinuing the application. However, no carcinomas were produced. Mody and Ranadive (1959) failed to elicit cancerous or precancerous changes in experimental mice by painting skin and buccal mucosa with alkaloid free extracts of tobacco. They observed only non-specific epithelial changes such as hyperplasia, thickening of horny layer and chronic inflammation in a small number of mice. However Reddy *et al.* (1960) successfully produced carcinomas in 80 per cent of mice treated with tobacco tar and heat.

### SUMMARY AND CONCLUSIONS

1. The paper presents a histological study of the human buccal mucosa exposed to betel quid chewing containing tobacco for a variable number of years. The biopsies were obtained from 3 groups of patients: (i) Tobacco chewers without clinical evidence of oral carcinoma (30 cases). (ii) Tobacco chewers with clinical evidence of oral carcinoma (30 cases). (iii) Control group of non-chewers and non-smokers without oral lesions (20 cases).

2. The commonest change observed among the group of chewers was hyperplasia of the epithelium. Parakeratosis was also a common finding among the chewers whereas hyperkeratosis was less commonly observed. Both these changes were distinctly rare among the non-chewer control cases.

3. The chief difference observed was in the incidence of dyskeratosis which is recognized as a premalignant change in the epithelium. Whereas it was observed in 6 and 11 cases in groups I and II respectively, it was not seen in a single instance in the non-chewer control cases. Also of importance was the finding of 2 cases of early invasive cancer in Group I patients, where clinically no malignancy was suspected. These observations support the view that tobacco plays an important role in the causation of oral cancer.

4. A diminution in the number of cases showing melanin pigment in the basal layer and subepithelial tissue was observed in chewers and was directly correlated with the intensity of epithelial change. This alteration probably represents a loss of specialized function following exposure to tobacco.

Thanks are due to Dr. P. D. Shroff for taking the biopsies. Without his co-operation this study would not have been possible.

### REFERENCES

Chapman, I. and Redish, C. H.—(1960) *Archs Path.*, **70**, 133.
Fredell, H. L. and Rosenthal, L. M.—(1941) *J. Am. med. Ass.*, **116**, 2130.
Helwig, F. C.—(1928) *J. Am. med. Ass.*, **91**, 150.
Jacobs, A.—(1960) *J. clin. Path.*, **13**, 463.
Mody, J. K. and Ranadive, K. J.—(1959) *Indian J. med. Sci.*, **13**, 1023.
Orr, I. M.—(1933) *Lancet*, ii, 575.
Padmavathy, G. and Reddy, D. J.—(1960) *J. Indian med. Ass.*, **34**, 84.
Paymaster, J. C.—(1956) *Cancer N.Y.*, **9**, 431.—(1957) *Br. J. Surg.*, **44**, 467.
Pindborg, J. J. and Poulsen, H. E.—(1962) *Acta path. microbiol. scand.*, **55**, 412.
Pindborg, J. J., Srivastava, A. N. and Gupta, D.—(1964) *Acta odont. scand.*, **22**, 499.
Reddy, D., Reddy, D. and Rao, P.—(1960) *Cancer, N.Y.*, **13**, 263.
Renstrup, G.—(1963) *Acta odont. scand.*, **21**, 333.
Sanghvi, L. D., Rao, K. C. M. and Khanolkar, V. R.—(1955) *Br. med. J.*, i, 1111.
Shanta, V. and Krishnamurthi, S.—(1963) *Br. J. Cancer*, **17**, 8.